# Importance of the early diagnosis of acromegaly. Evolution of mandibular growth and dental occlusion in a case with a late diagnosis

**DOI:** 10.4317/jced.63473

**Published:** 2025-11-30

**Authors:** Jordi Borrás-Ferreres, Severino Boluda-Monzo, Cosme Gay-Escoda

**Affiliations:** 1 DDS, MSc. Private practice in Oral Surgery and Implantology, Benicarló (Castellón), Spain; 2 MD, MSc. Head of the Department of Endocrinology, Vinaroz Regional Hospital, Vinaroz (Castellón), Spain; 3 MD, DDS, MSc, PhD, EBOS, OMFS. Chairman and Professor of the Department of Oral and Maxillofacial Surgery, Faculty of Medicine and Health Sciences, School of Dentistry, Uni¬versity of Barcelona. Director of the Master degree program in Oral Surgery and Implantology, EFHRE International University/FUCSO, Barcelona. Founder/Researcher of the IDIBELL Institute. Head of the Department of Oral and Maxillofacial Surgery and Implantology, Teknon Medical Center, Barcelona, Spain

## Abstract

**Background:**

Despite the high prevalence of oral and maxillofacial manifestations in the early stages of acromegaly, dentists do not represent an important percentage of the healthcare professionals that diagnose the disease.

**Case Report:**

The present study describes the buccodental and facial changes occurring over a period of 20 years in a patient with a late diagnosis of acromegaly.

**Discussion:**

The facial disfiguration and buccodental problems, particularly mandibular enlargement and malocclusion, may be the key elements leading to the diagnosis.

**Conclusions:**

It is essential to enhance awareness of the disease among dentists, and particularly among orthodontists, as they are ideally positioned to recognize and diagnose acromegaly in its early stages.

## Introduction

Acromegaly is a rare chronic disorder caused by an excessive secretion of growth hormone (GH), in most cases secondary to the presence of a hypophyseal (pituitary gland) adenoma that leads to an increase in the blood concentration of insulin-like growth factor 1 (IGF-1), the main molecule responsible for the activity of GH (1). In contrast to gigantism, acromegaly develops once skeletal development has already ended and epiphyseal closure has been completed. Consequently, the hormone only causes uncontrolled growth of the facial bones and mandible, producing facial disfiguration and alterations in dental occlusion, together with enlargement of the acral (outermost or distal) parts of the body (2 - 4).

The prevalence of acromegaly is 20-130 cases per million people, with an annual incidence of 2-11 cases per million individuals. The mean patient age at the time of diagnosis is 40-50 years (1).

Unfortunately, the delay in diagnosis ranges between 5 and over 10 years after the onset of the disease, and during this period patients experience progressive worsening of their symptoms and quality of life (1 , 3 , 5). This delay is explained by the insidious nature of the disease, starting with mild and nonspecific signs and symptoms which neither the patient nor the physician attribute to any specific disease process (3).

Since the buccodental alterations and facial changes appear at an early stage, dentists, and especially orthodontists, should be able to reliably recognize these early manifestations of acromegaly (4 , 6). In many cases this would probably allow an earlier diagnosis of the disease, thus substantially improving the prognosis and quality of life of the patients (1 , 5).

The present study describes the buccodental and facial changes occurring over a period of 20 years in a patient with a late diagnosis of acromegaly, seeking to increase awareness of the disease among dentists, and particularly orthodontists.

## Case Report

After 15 years without follow-up, a 36-year-old male consulted for possible orthognathic surgical treatment that had already been recommended on occasion of his last visit, in 2009. Over time, his malocclusion had worsened, affecting his self-esteem, chewing capacity and quality of life.

**Figure 1:**
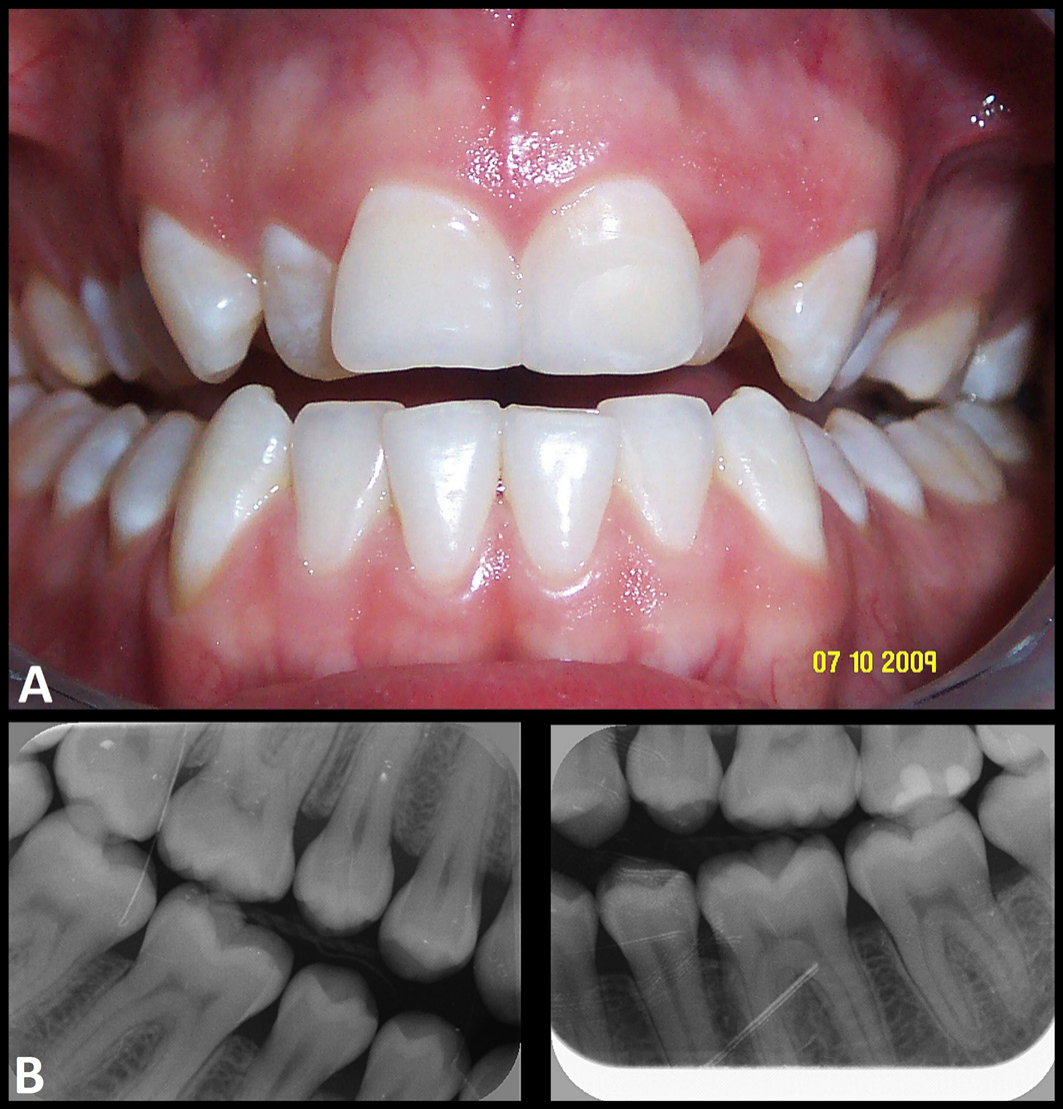
Images of the patient in 2009, at 21 years of age. A. Dental occlusion. B. Bite-wing radiographic views.

In 2009, when the patient was 21 years old, he had been diagnosed with dental malocclusion with anterior and posterior open bite - the only interocclusal contacts being limited to the second and third molars (Fig. 1).

1

**Figure 2:**
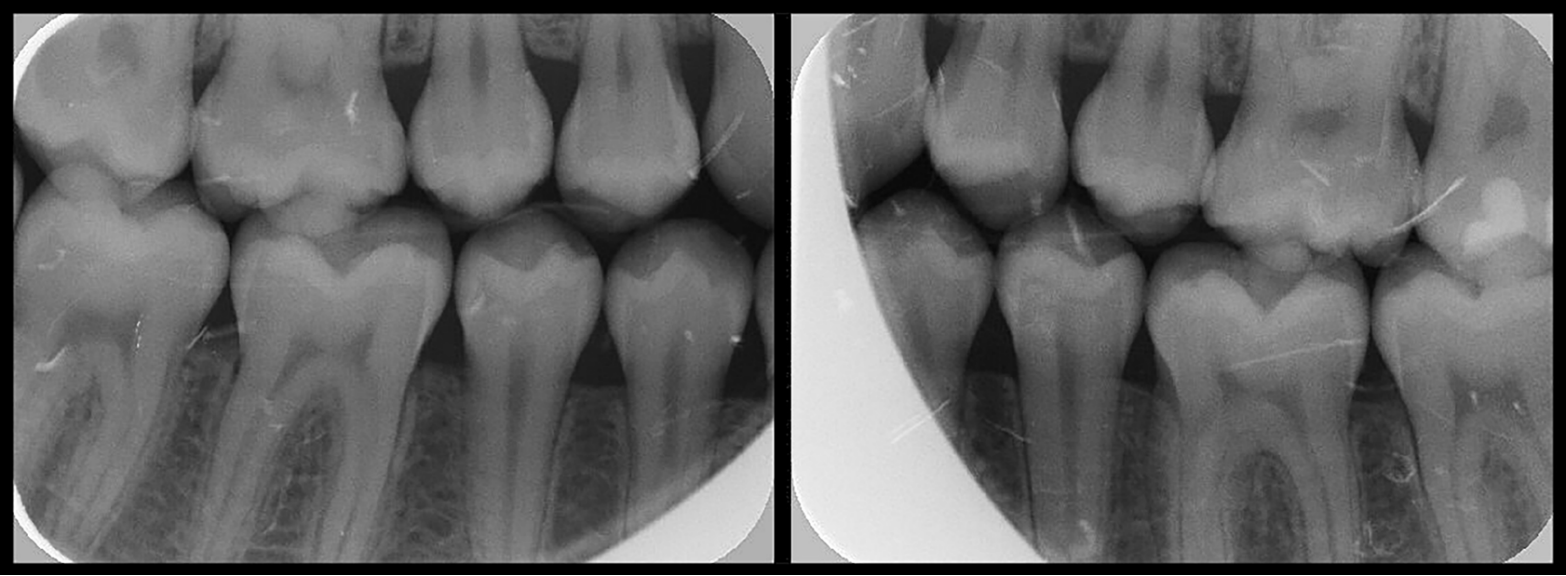
Bite-wing radiographic views of the patient in July 2005, at the age of 17 years.

At that time, orthognathic surgery combined with orthodontic management was proposed, but was finally rejected by the patient. It should be noted that four years earlier, at 17 years of age and still in the active growth phase, his dental occlusion was normal and he was receiving no pharmacological treatment (Fig. 2).

2

**Figure 3:**
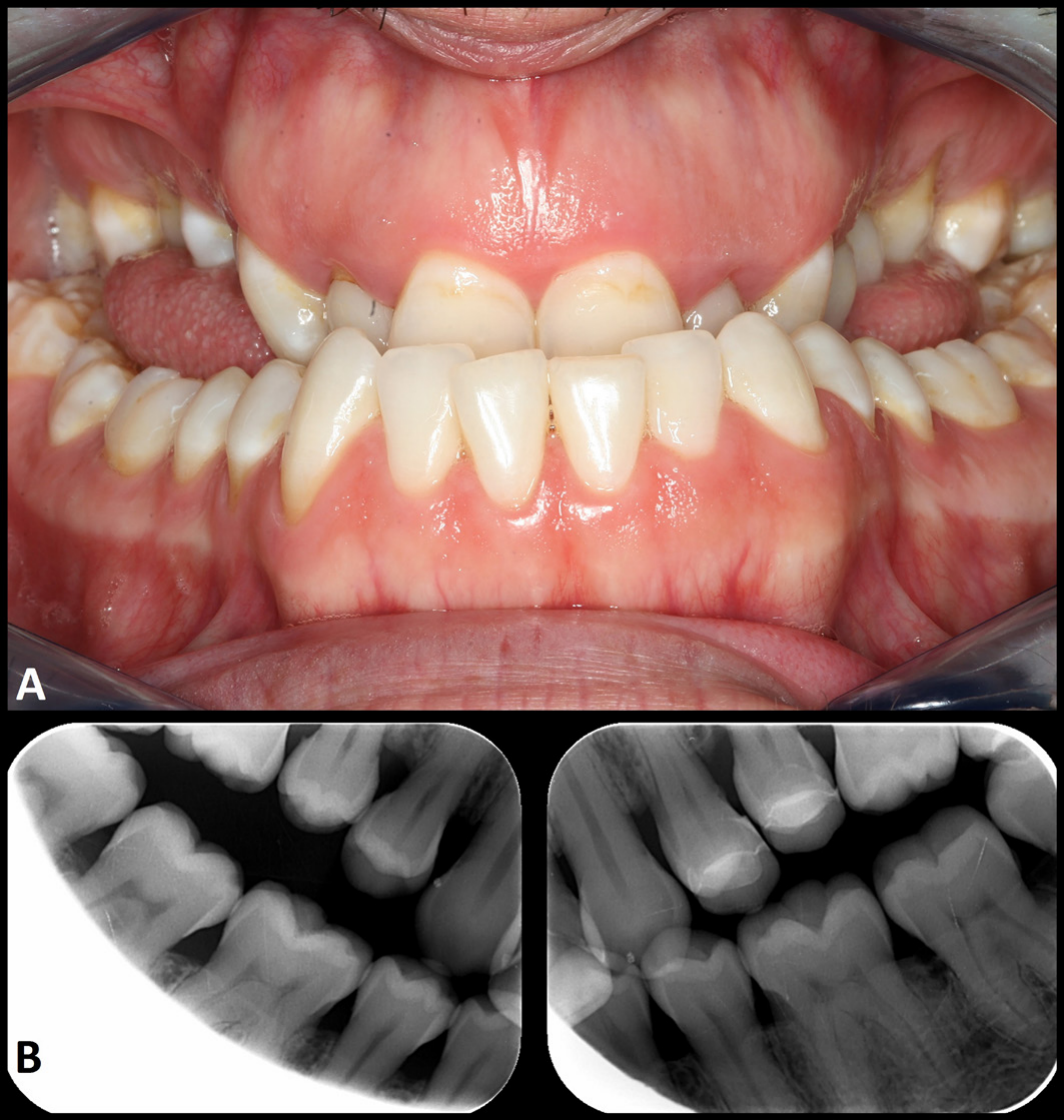
Views of the patient in June 2024, at the age of 36 years. A. Dental occlusion. B. Bite-wing radiographic views.

His current (2024) facial appearance came as a surprise to the professional team of the clinic, as it had changed notoriously since the last visit 15 years ago, with clear mandibular prognathism being the most significant observation. The oral examination revealed important skeletal class 3 malocclusion, with anterior cross-bite and open bite in the posterior areas, together with macroglossia (Fig. 3).

3

**Figure 4:**
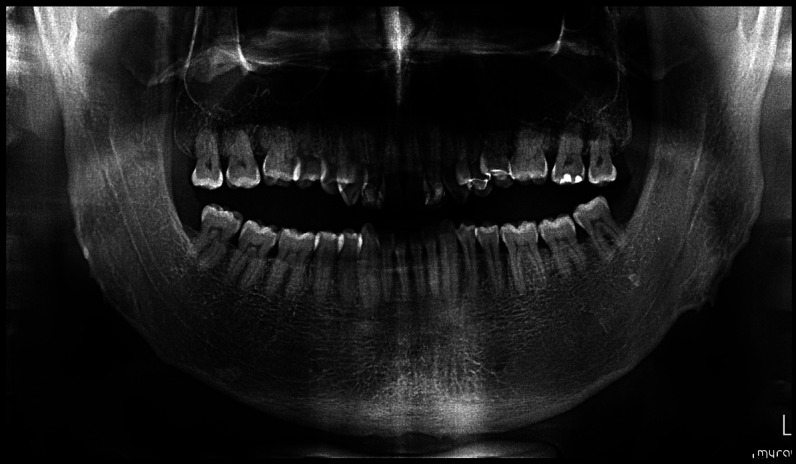
Panoramic radiographic view of the patient in June 2024.

The panoramic radiographs showed very obtuse gonial angles and a large body of the mandible and ascending rami (Fig. 4).

4

The evolution over the last 15 years, with facial disfiguration and the oral findings, suggested possible acromegaly. Subsequent questioning revealed that the patient had noticed enlargement of his feet over time, causing him to increase the size of his footwear, and he moreover suffered sleep disturbances.

The laboratory tests showed blood GH 34.20 ng/ml and IGF-1 1117 ng/ml. In view of the hormonal alteration, a magnetic resonance imaging (MRI) study of the hypothalamic-pituitary region was performed before and after gadolinium contrast injection. The pituitary was seen to be enlarged due to the presence of a focal lesion that occupied the entire gland, measuring about 17 x 12 mm, and which showed hypovascularization following contrast administration. There was no evidence of invasion of neighboring structures. With an initial diagnosis of pituitary adenoma, the patient was referred to the Neurosurgery Department, where transnasal removal of the tumor was carried out, and the initial diagnosis was confirmed. One month after surgery, the blood GH concentration had decreased to 0.09 ng/ml and IGF-1 to 149 ng/ml. As a complication of the operation, the patient developed secondary hypopituitarism that was treated with desmopressin, levothyroxine, hydrocortisone and testosterone cyclopentylpropionate.

## Discussion

Acromegaly is a rare endocrine disease with an incidence of about 5 cases per million individuals and a worldwide prevalence of approximately 60 cases per million individuals (3 , 7 , 8). Most studies have reported no gender differences (7 , 9), although some publications have described a slightly higher prevalence in women (10). The mean patient age at the time of diagnosis is 40-50 years (9 , 11 , 12), with a more aggressive presentation of the disease in younger patients (13). The cause of acromegaly is an overproduction of GH and high IGF-1 levels, in most patients secondary to a pituitary gland adenoma (1 , 3). A recent meta-analysis of 16 studies confirmed a global 72% increase in mortality among patients with acromegaly compared with the general population (14). The main causes of death in these individuals are cardiovascular problems that develop over time, fundamentally arterial hypertension, acromegalic cardiomyopathy, coronary artery disease, valve disorders and arrhythmias (15 , 16). The most common manifestations include disproportionate skeletal growth, particularly of the face, hands and feet (1 - 4 , 11). It is therefore not surprising that patients often report having to enlarge their ring size or the size of their footwear over time (11). At facial level, growth of the glabellar zone of the frontal bone and especially of the mandible, along with thickening of the soft tissues (nose, lips and skin), result in a coarse facial appearance (2 - 4). According to De Stefani et al. (1), mandibular growth and prognathism, with consequent skeletal class 3 malocclusion, is the most significant facial change, as seen in our patient. This condition appears to be caused by reactivation of the cartilage cells of the mandibular condyles, under the stimulation of IGF-1 (3). Unfortunately, the obtainment of clinical images was rejected by the patient, so we have no photographs showing this. The most frequent patient complaints are headache, increased perspiration, acroparesthesias and joint pain (1 , 11 , 17). The suspicion of acromegaly must be confirmed by blood tests, including the determination of GH and IGF-1 levels following an oral glucose tolerance test, as well as a magnetic resonance imaging (MRI) study of the pituitary gland (11). Despite the existence of clear signs and symptoms, most patients suffer a delay in diagnosis of about 5-10 years (1 , 3 , 5). This delay is associated with the development of many weakening comorbidities such as obstructive sleep apnea, arterial hypertension, biventricular hypertrophy, diabetes mellitus, dyslipidemia, hypercalcemia, hyperuricemia or osteoporosis (2 - 4 , 16). These problems logically result in a series of psychosocial complications such as anxiety and depression (5), as in our patient. There are different treatment options for pituitary adenoma, including resection of the tumor (first-line treatment), as in our case, or pharmacological and/or radiotherapeutic management in patients with an important surgical risk or in whom surgery has proved ineffective. The aim is to control GH and IGF-1 secretion, and the comorbidities, as well as reduce mortality, and restore patient quality of life (18). Although in our patient the GH and IGF-1 levels could be controlled as a result of transsphenoidal removal of the adenoma, the surgical damage produced hypopituitarism requiring hormone replacement therapy. In addition to the facial changes and growth of the extremities, up to 80% of all patients with acromegaly have buccodental manifestations, mainly mandibular growth (22-24%) and highly variable occlusal alterations - including skeletal class 3 malocclusion (20-22%) (2 - 4 , 9 , 17), diastemas (40-43%) (3 , 9 , 11) and macroglossia (54-58%) (3 , 4 , 9). Kreischmann-Andermahr et al. (3), in a study of 145 patients with acromegaly, reported buccodental alterations in 80.7% of the cases, particularly enlargement of the tongue (57.9%), diastemas (42.8%), mandibular growth (24,1%) and mandibular prognathism (22.1%). Although occlusal alterations in the lateral zones are rare, they did appear in our patient. Furthermore, the macroglossia and pharyngeal thickening often seen in patients with acromegaly are often accompanied by a hoarse and deep voice, together with the presence of obstructive sleep apnea, as was also noted in our patient (3 , 4 , 9 , 19). Table 1 summarizes the main oro-maxillo-facial features of acromegaly and the possible non-endocrine treatment options (1 - 3 , 6 , 11 , 17 , 20 , 21).

Table 1

However, despite the high prevalence of buccodental manifestations in acromegaly, dentists and orthodontists do not play a key role in suspecting and diagnosing the disease, despite the fact that they typically document their patients with photographs and dental radiographs in routine practice (6). In a recent study by the Liège Acromegaly Survey (22), with the participation of over 3000 patients, acromegaly was found to be identified most often by endocrinologists (44.9%), general practitioners / family physicians (17.5%) or internists (13.2%), with a lesser involvement of rheumatologists / orthopedists (3.6%), neurologists (3.3%) and ophthalmologists (2.3%). The range of non-endocrinological specialists that diagnose acromegaly is significant, though dentists do not appear to play an important role in this regard, as reflected by other studies (23 - 25). The study carried out by Preo et al. (6) involving 426 dentists, including 206 orthodontists, reported that only 10% of them suspected the disease in a group of patients. As mentioned, facial disfiguration and buccodental problems, particularly mandibular enlargement and malocclusion, may be the basic patient features leading to the diagnosis, and are moreover correlated to the duration of the disease (3 , 4 , 6). Kreitschmann-Andermahr et al. (3), in 145 patients with acromegaly, found an early diagnosis within the first two years to be significantly associated with a lesser incidence of oral problems than when the diagnosis was established in a later stage (68.5% versus 87.2%), and patient quality of life was moreover better (p = 0.014). We consider it essential for dentists, and particularly orthodontists, to be aware that the oral manifestations develop early and are often the first signs of the disease - placing these professionals in an excellent position for establishing an early diagnosis. However, as evidenced by Preo et al. (6), the fact that only a very small percentage of these specialists report having suspected the disease suggests that they still do not have enough knowledge and sensitivity in relation to acromegaly. Dentist awareness of the clinical features of patients with acromegaly, the use of photographs with facial and oral details of the patients, and radiographic follow-up, could help establish an earlier diagnosis of the disease. This may not only reduce or even prevent greater facial disfiguration but also improve patient wellbeing, since disfiguration is the most disabling problem for these individuals, with an impact upon self-esteem, body image and quality of life (3 , 5). Unfortunately, in our patient the late diagnosis was attributable to the fact that he had not been seen for 15 years, since he failed to report to the scheduled follow-up visits. However, on a retrospective basis, we already should have suspected acromegaly back in 2009, when the disorder had started to show its first buccodental manifestations. This unfortunate error and its dramatic consequences led us to publish the present study.

## Conclusions

Despite the high prevalence of oral and maxillofacial manifestations in the early stages of acromegaly, dentists do not represent an important percentage of the healthcare professionals that diagnose the condition. It is essential to enhance awareness of the disease among dentists, and particularly among orthodontists, as they frequently conduct radiographic and photographic controls that can be contrasted over time, with the specific aim of recognizing and diagnosing acromegaly in its early stages. This would have a significant impact upon the prognosis and quality of life of the patients.

## Figures and Tables

**Table 1:** Oro-maxillo-facial features of acromegaly and possible treatments [1].

Symptoms/Signs	Diagnosis	Possible treatments
Class III malocclusion	Dental evaluation	No treatment or orthodontic treatment—if necessary, orthodontic-surgical treatment—in the inactive phase of the disease
Dental diastema	Dental evaluation	Possible conservative treatment in any phase of thedisease, or orthodontic treatment, preferably not in theactive phase of the disease
Macroglossia	Mallampati or Modified-Mallampati evaluation	Medical management for the control of acromegalicsyndrome; possible surgical or conservative treatment ofobstructive sleep apnea syndrome with special dental appliances
Osseous tori or exostoses	Dental evaluation	No treatment; surgical treatment in the event that theexostoses hinder the insertion of dental prostheses
Incongruity of mobile prostheses or implant-based prostheses, and possible breakage	Dental evaluation	Adjustment of the prosthetic bases or replacement of thesame; in the event of prostheses fixed on implants,verification and possible adjustment of the intraosseouspillars, preferably not in the active phase of the disease
Inclination of frontal elements	Orthodontic evaluation	No treatment, or orthodontic treatment in the inactivephase of the disease
Hypercementosis	Fortuitous, following routineradiographic examinations	No treatment
Temporomandibular joint clicks and pain	Dental and maxillaryevaluation, radiographicdeepening with MRI	Functional treatment with gymnastics for thetemporomandibular joint or application of a customizedbite.BruxApp can be adopted under the control ofthe dentist to monitor and correct incorrect or flawedpositions maintained during the day by the patient, andto guide postural re-education
Thickening of gingival tissues	Dental evaluation	No treatment, or surgical treatment in the inactive phaseof the disease

1

## Data Availability

The datasets used and/or analyzed during the current study are available from the corresponding author.
